# Amphiregulin and PTEN evoke a multimodal mechanism of acquired resistance to PI3K inhibition

**DOI:** 10.18632/genesandcancer.10

**Published:** 2014-03

**Authors:** Kyle A. Edgar, Lisa Crocker, Eric Cheng, Marie-Claire Wagle, Matthew Wongchenko, Yibing Yan, Timothy R. Wilson, Nicholas Dompe, Richard M. Neve, Marcia Belvin, Deepak Sampath, Lori S. Friedman, Jeffrey J. Wallin

**Affiliations:** ^1^ Departments of Translational Oncology, South San Francisco, CA, USA; ^2^ Department of Oncology Biomarkers, South San Francisco, CA, USA; ^3^ Department of Molecular Biology, Genentech, Inc., South San Francisco, CA, USA

**Keywords:** PI3K, drug resistance, PTEN, amphiregulin

## Abstract

Phosphoinositide-3 kinase (PI3K) signaling pathway alterations occur broadly in cancer and PI3K is a promising therapeutic target. Here, we investigated acquired resistance to GDC-0941, a PI3K inhibitor in clinical trials. Colorectal cancer (CRC) cells made to be resistant to GDC-0941 were discovered to secrete amphiregulin, which resulted in increased EGFR/MAPK signaling. Moreover, prolonged PI3K pathway inhibition in cultured cells over a period of months led to a secondary loss of PTEN in 40% of the CRC lines with acquired resistance to PI3K inhibition. In the absence of PI3K inhibitor, these PTEN-null PI3K inhibitor-resistant clones had elevated PI3K pathway signaling and decreased sensitivity to MAPK pathway inhibitors. Importantly, PTEN loss was not able to induce resistance to PI3K inhibitors in the absence of amphiregulin, indicating a multimodal mechanism of acquired resistance. The combination of PI3K and MAPK pathway inhibitors overcame acquired resistance in vitro and in vivo.

## INTRODUCTION

The phosphatidylinoisitol 3'-kinase (PI3K) signaling pathway can be activated by a variety of extracellular signals and is involved in cellular processes such as survival, proliferation, migration and protein synthesis [1]. Aberrant activation of this pathway has been widely implicated in cancers. Two major hot spot mutations in the PI3K catalytic subunit have been reported, one in the helical domain (E545K) and the other in the kinase domain (H1047R). Both mutations are transforming and result in increased pathway signaling [2-4]. The tumor suppressor protein phosphatase and tensin homologue (PTEN) acts to inhibit PI3K pathway signaling and is commonly mutated, deleted or epigenetically repressed in human cancers [5, 6]. Due to the dysregulation of the PI3K pathway in many cancers, there are increasing efforts in the development of PI3K pathway inhibitors as potential therapeutics with reports of efficacy being reported [7]. Although PI3K inhibitors offer an additional line of treatment, as with other targeted therapies, acquired resistance is likely to arise.

To investigate resistance to PI3K inhibitors, it is important to examine mechanisms that are upstream of PI3K signaling. The PI3K pathway can be activated by mutations or overexpression of upstream signaling molecules in the ErbB family of receptor tyrosine kinases, such as EGFR/ErbB1, HER2/ErbB2, and HER3/ErbB3 [8-11]. EGFR ligands bind and activate the EGF receptor and include EGF, amphiregulin (AREG), βcellulin (BTC), epiregulin (EPR), transforming growth factor α (TGFα), heparin-binding EGFR-like growth factor (HB-EGF), and epigen [12]. The activation of EGFR is prevalent in cancer signaling and not only activates PI3K by recruiting the regulatory subunit, p85 [13], but also induces activation of the mitogen-activated protein kinase (MAPK) pathway by either Grb2 or Shc adaptor proteins [14]. EGFR signaling has been implicated as a mechanism of resistance to several targeted cancer therapies, such as crizotinib [15], trastuzumab [16, 17], and vemurafenib [18]. Not only has dysregulation of EGFR conferred drug resistance, but stimulation by EGF ligands has been shown to subvert inhibition of targeted inhibitors as well [19].

Despite the amount of activity in the development of PI3K inhibitors, less is known about acquired resistance to these inhibitors. Engineered mouse models that express an activating H1047R mutation in PIK3CA have found up-regulation of c-Myc to be involved in PI3K inhibitor resistance [20]. In these studies, MET amplified tumors remained dependent on endogenous PI3K, while c-Myc amplified tumors became pathway independent. Additional studies using engineered cancer cells have also identified increases in c-Myc as well as eI4FE and Notch1 as potential mechanisms of resistance [21, 22].

GDC-0941 is an orally bioavailable inhibitor of Class I PI3K that is in clinical development for several solid tumor indications [23-25]. In these studies we investigate mechanisms of resistance to GDC-0941 in the SW48 CRC line that is wild-type for PI3Kα or harbors an oncogenic H1047R PI3Kα mutation. Parental SW48 and SW48 H1047R cells are able to overcome growth suppression by GDC-0941 by the addition of EGFR ligands. In addition, SW48 cell lines that have acquired resistance to GDC-0941 initiate secretion of the EGFR ligand AREG, which allows the cells to continue to grow and survive in the presence of GDC-0941. We also found that resistant cells lose PTEN after long-term culture, thereby increasing PI3K pathway signaling. These results may provide guidance on potential clinical treatment regimens.

## RESULTS

### EGFR ligands confer resistance to GDC-0941 in SW48 isogenic cells

A CRC cell line, SW48, and a version of this cell line with a knock-in H1047R PI3Kα mutation at one of the endogenous loci were used to investigate cellular changes associated with oncogenic PI3K. The introduction of the H1047R mutation to the SW48 cell line resulted in increased cell growth and increased PI3K pathway signaling as measured by pAKT^T308^, pAKT^S473^, pPRAS40^T246^, pp70S6^T389^, and pS6^S235/236^ (Supplemental Figure 1A and [26]. We found the parental and PI3K mutant cell lines were sensitive to the PI3K inhibitor, GDC-0941 (Supplemental Figure 1B).

**Figure 1:**
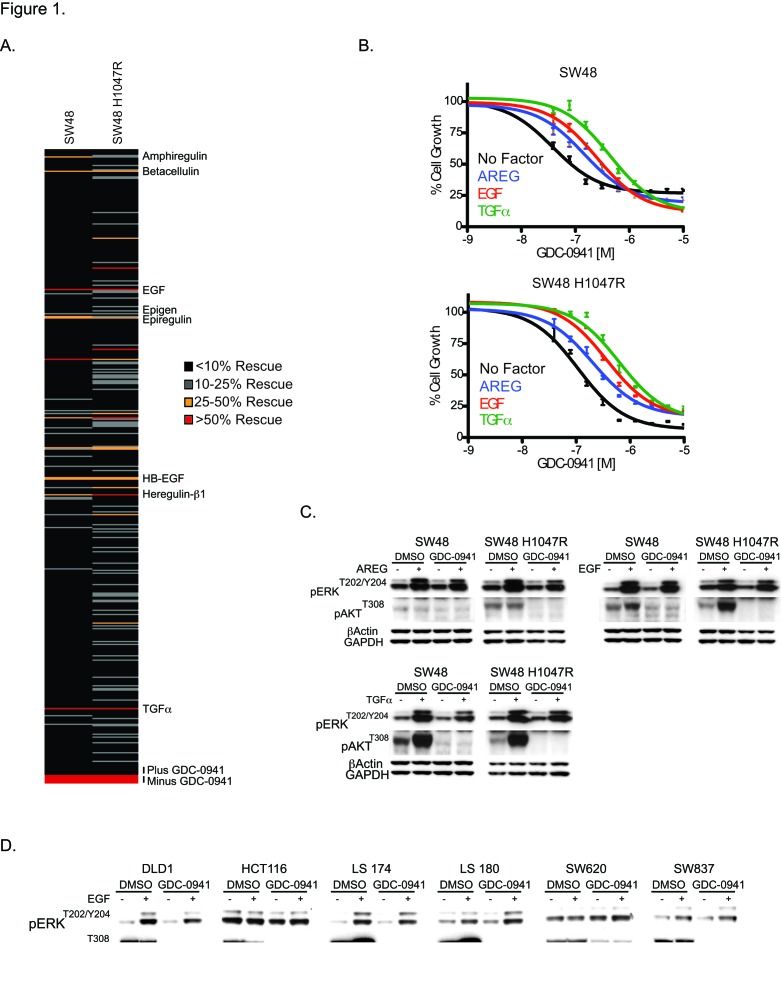
Characterization of the effects of secreted ligands on GDC-0941 potency. (A) SW48 and SW48 H1047R cells treated with DMSO, GDC-0941 alone, or GDC-0941 plus 50 ng/ml of one of 418 secreted ligands, and assayed for viability. Controls in the absence of secreted factors and in the presence or absence of GDC-0941 are shown at bottom. (B) SW48 and SW48 H1047R cells treated with GDC-0941 alone, or GDC-0941 plus 50 ng/ml of AREG, EGF, or TGFα, and assayed for viability. (C) SW48 and SW48 H1047R cells simulated with 50 ng/ml of AREG, EGF, or TGFα and treated with either DMSO or 1.5 uM of GDC-0941. After 1 hr, cell lysates were prepared and analyzed by immunoblotting for pERK^T202/Y204^ and pAKT^T308^. (D) Six colorectal cancer cell lines simulated with 50 ng/ml of EGF and treated with either DMSO or 1.5 uM of GDC-0941. After 1 hr, cell lysates were prepared and analyzed by immunoblotting for pERK^T202/Y204^ and pAKT^T308^.

To investigate the potential role of soluble ligands in resistance to GDC-0941 we utilized a screen of commercially available factors to identify candidates that rescue GDC-0941-induced growth inhibition. For the screen, SW48 and SW48 H1047R cells were dosed with a 90% maximum inhibitory concentration (IC_90_) of GDC-0941 (1 uM) as well as 50 ng/ml of one of 418 soluble ligands for 72 hours (Supplemental Table 1). We found 11 factors (3% of total) were able to rescue GDC-0941-induced growth inhibition greater than 25%. Of the 11 factors, 8 belonged to the epidermal growth factor receptor (EGFR) ligand family: AREG, Bcellulin, EGF, Epigen, Epiregulin, HB-EGF, HRG-11, and TGFα (Figure 1A). To confirm the ability of EGFR ligands to overcome GDC-0941 growth inhibition, the EGFR ligands AREG, EGF and TGFα were tested for their effect on GDC-0941 cellular potency. In the SW48 line, AREG, EGF, and TGFα were able to decrease GDC-0941 sensitivity, 2.5-fold, 4.2-fold, and 7.5-fold respectively (Figure 1B). The effects were comparable in SW48 H1047R cells, where GDC-0941 inhibition was reduced 2-fold by AREG, 3.3-fold by EGF, and 5.2-fold by TGFα.

To find the underlying mechanism of EGFR ligand ability to decrease GDC-0941 sensitivity, we investigated downstream signaling in the PI3K and MAPK pathways. All three ligands tested (AREG, EGF, and TGFα were shown to increase pERK1/2^T202/Y204^ in both cell lines irrespective of the presence of GDC-0941, suggesting the cells could use MAPK pathway activation under conditions of ligand stimulation (Figure 1C).

To confirm this effect was not specific to the SW48 line, an additional 6 colorectal cancer cell lines were screened for the ability of EGF to negatively impact the cellular potency of GDC-0941. Of the 7 total lines (including SW48), 5 showed decreased sensitivity to GDC-0941 in the presence of EGF that was greater than 1.7-fold (Supplemental Table 2). We investigated PI3K and MAPK pathway signaling in these cell lines. In the two lines where GDC-0941 potency was minimally influenced by EGF (HCT116 and SW620), stimulation with EGFR ligands (AREG, EGF, and TGFα did not effect levels of pERK1/2^T202/Y204^ in the presence of GDC-0941 (Supplemental Table 2, Figure 1D and Supplemental Figures 2A and 2B). It is important to note that of the 6 additional cell lines only SW620 did not have detectable levels of EGFR by western blot (Supplemental Figure 2C). This suggested that these cells could not use MAPK pathway activation under EGFR ligand stimulation conditions. The four cell lines where GDC-0941 potency was most affected by the presence of EGF were shown to increase pERK1/2^T202/Y204^ irrespective of the presence of GDC-0941, suggesting the cells could activate the MAPK pathway under conditions of EGFR ligand stimulation. The results in these four cell lines are similar to those observed with SW48 cells.

### GDC-0941 resistant cells exhibit increased PI3K pathway signaling when GDC-0941 is removed

**Figure 2:**
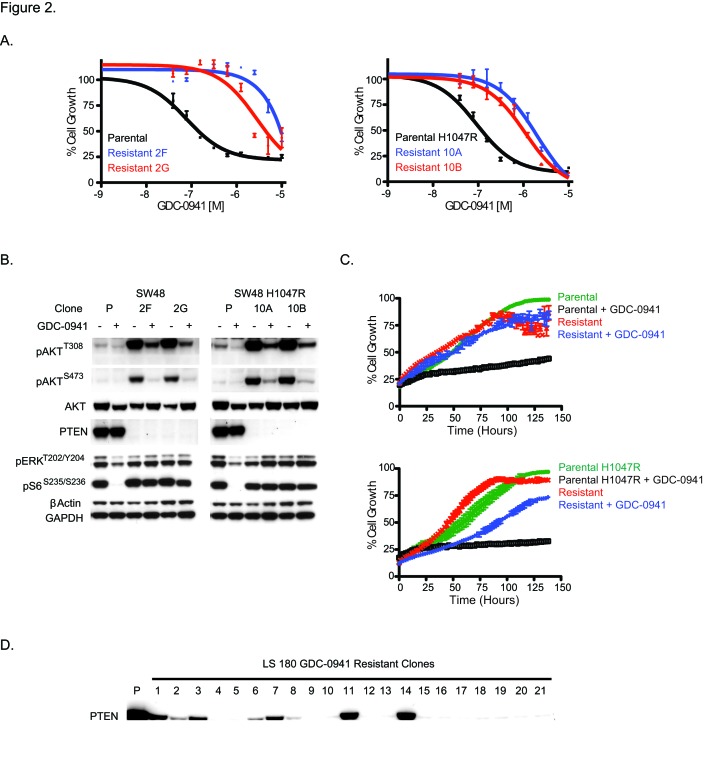
Characterization of SW48 GDC-0941 resistant clones. (A) SW48 and SW48 H1047R GDC-0941 parental and resistant clones treated with a dose escalation of GDC-0941 and assayed for viability 96 hrs post dosing. (B) GDC-0941 parental (P) and resistant clones grown for 48 hrs either in the presence of DMSO or 1.5 uM of GDC-0941. After 48 hrs, cell lysates were prepared and analyzed by immunoblotting. (C) GDC-0941 parental and resistant clones (2F top, 10A bottom) were plated at 15,000 cells/ well and allowed to adhere overnight. The following day, cells were treated with DMSO or 1.5 uM of GDC-0941 and percent confluence was monitored by digital imaging. (D) LS 180 parental (P) and 21 GDC-0941 resistant clones analyzed by immunoblotting for PTEN protein.

In addition to investigating the role of EGFR ligands in acute or innate PI3K inhibitor resistance, we sought to examine factors involved in acquired GDC-0941 resistance. Pools of SW48 or SW48 H1047R cells were treated at increasing doses of GDC-0941 over 6 months. At the end of dose escalation, the cells were able to grow at a GDC-0941 concentration 10-fold higher (1.5 uM) than the initial EC_50_ dose (0.15 uM). In a viability assay, the SW48 resistant pool was 10-fold less sensitive than the parental SW48 cell line to GDC-0941 while the SW48 H1047R resistant pool was 13-fold less sensitive than parental SW48 H1047R cells (Supplemental Figure 1B). Clones from GDC-0941 resistant pools were generated by plating single cells of each line in the presence of 1.5 uM GDC-0941 and isolating proliferating clones. Two resistant clones of each cell type were assessed and characterized for SW48 (clones 2F and 2G) and SW48 H1047R (clones 10A and 10B). SW48 resistant clones 2F and 2G had a 120- and 36-fold decrease in GDC-0941 sensitivity, respectively, compared to the SW48 parental line. SW48 H1047R resistant clones 10A and 10B had a 22- and 12-fold decreased sensitivity to GDC-0941, respectively, compared to the SW48 H1047R parental line (Figure 2A). To confirm that the observed resistance was not specific to GDC-0941, the potency of another PI3K inhibitor, GDC-0980 (27), was evaluated in resistant clones. We found the resistant clones were also not sensitive to GDC-0980 (Supplemental Table 3).

Once resistant pools and clones were confirmed to retain resistance to GDC-0941, signaling downstream of PI3K was assessed by reverse phase protein array (RPPA) and western blot. Both resistant pools and all four resistant clones were shown to have increased levels of pAKT at both the T308 and S473 phosphorylation sites, which was strongly enhanced when clones were released from the 1.5 uM dose of GDC-0941 (Figure 2B, Supplemental Figure 3). Further exploration of PI3K pathway members revealed that all resistant cell lines had lost the tumor suppressor, PTEN, which helps to explain the observed pAKT increase. The PTEN protein loss observed by western blot was confirmed with a PTEN expression decrease by microarray (Supplemental Figure 4). To assess how these cells grow due to PTEN loss, cells were imaged every 4 hours for 140 hours. In the SW48 line, the parental line did not grow in the presence of GDC-0941 while the resistant clone grew at the same rate as the parental line in the presence or absence of GDC-0941 (Figure 2C). Comparably, the SW48 H1047R parental line was growth arrested in the presence of GDC-0941, while the SW48 H1047R resistant clone grew in the presence of GDC-0941 (Figure 2C). While the loss of PTEN and corresponding increase of phosphorylated AKT may play a role in the resistant clones becoming insensitive to GDC-0941, we also found muted GDC-0941 responses in clones with respect to both pERK1/2^T202/Y204^ and pS6^S235/236^ when compared to sensitive clones, suggesting alternate pathways may be playing a role in the resistance (Figure 2B). ERK Inhibition has been observed with other PI3K inhibitors, but the mechanism is unknown (28).

To confirm that loss of PTEN was not exclusive to SW48 cell lines, four additional colorectal CRC lines, DLD-1, HCT-116, LS 180, and SW620 were made resistant to GDC-0941 and cloned as described for SW48 cells (Supplemental Figure 5A). All clones were assessed for PTEN protein expression (Figure 2D and Supplemental Figure 5B). We discovered that 13 out of 21 LS 180 resistant clones had PTEN protein absent. GDC-0941 resistant clones from DLD-1, HCT-116, and SW620 lines expressed normal PTEN protein levels.

### GDC-0941 resistant clones secrete AREG that activates the MAPK pathway in the presence or absence of GDC-0941

**Figure 3:**
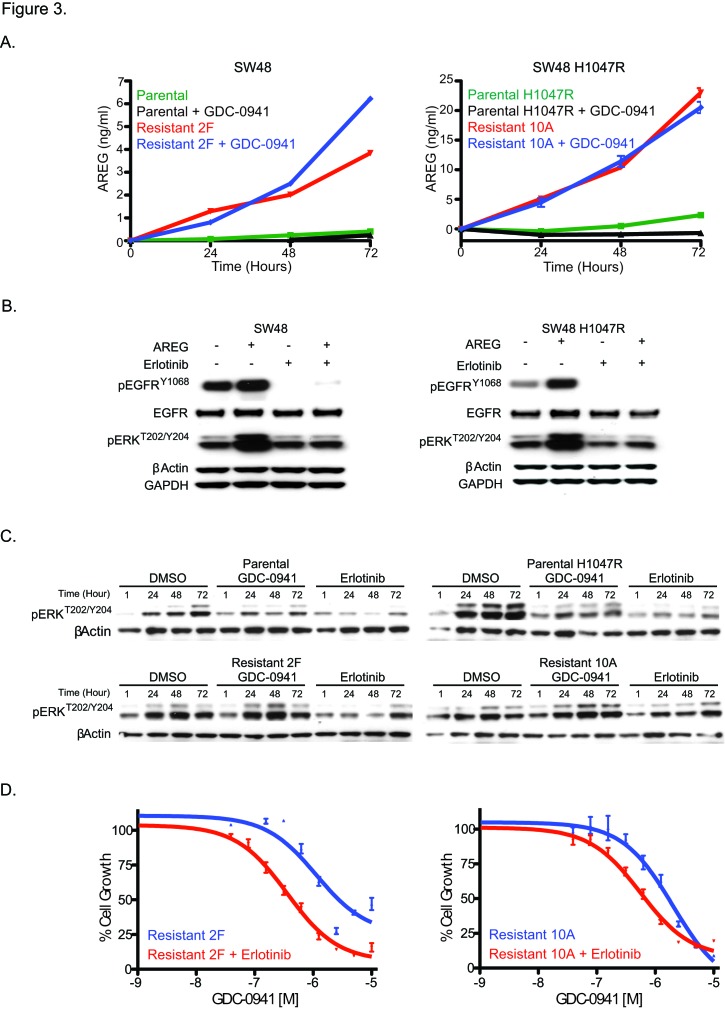
GDC-0941 resistant clones secrete AREG and can be re-sensitized by EGFR inhibition. (A) Media collected from SW48 and SW48 H1047R GDC-0941 parental and resistant clones assessed for AREG levels after treatment with DMSO or 1.5 uM of GDC-0941 at 24, 48, and 72 hrs post treatment. (B) Parental cells simulated with 50 ng/ml of AREG, treated with either DMSO or 1.0 uM of erlotinib for 1 hr. After 1 hr, cell lysates were prepared and analyzed by immunoblotting for the markers indicated. (C) SW48 and SW48 H1047R parental and GDC-0941 resistant clones dosed with DMSO, 1.5 uM of GDC-0941 or 1 uM of erlotinib and collected at 1, 24, 48, and 72 hrs post treatment. Cell lysates were prepared and analyzed by immunoblotting for pERK^T202/Y204^. (D) SW48 and SW48 H1047R GDC-0941 resistant clones were treated with a dose escalation of GDC-0941 with and without a constant 1 uM dose of erlotinib and assayed for viability 96 hrs post dosing.

Since EGFR ligands were able to overcome GDC-0941 sensitivity in both SW48 and SW48 H1047R cell lines (Figure 1B), resistant clones from these lines were assayed for a possible increase in EGFR ligand secretion. Levels of AREG, βcellulin, HRG-β1, EGF, HB-EGF, and TGFα were all assayed in resistant clone media and only AREG was detected. AREG levels in cell supernatants increased over time and were unchanged with PI3K inhibition in resistant clones, while no increase was observed in parental lines (Figure 3A). We previously discovered that AREG was able to activate the MAPK pathway in SW48 and SW48 H1047R cells in the presence of GDC-0941 (Figure 1D). Treatment of the cells with erlotinib under these conditions, however, was able to block signaling to pERK1/2^T202/Y204^ (Figure 3B). MAPK signaling was assessed in parental and resistant clones in normal cell culture conditions and we found that pERK1/2^T202/Y204^ increased over time, likely due to entry of cells into the cell cycle after plating (Figure 3C). A dose of 1.5 μM of GDC-0941 prevented the pERK1/2^T202/Y204^ increase in parental lines, while it had a muted effect in resistant clones, but erlotinib was able block the increase in all lines (Figure 3C). Resistant clones were assayed for GDC-0941 sensitivity with and without the presence of erlotinib (Figure 3D and Supplemental Figure 7A). Sensitivity to GDC-0941 was increased by addition of erlotinib 3.2-fold in SW48-R clone 2F and 3.6-fold in SW48 H1047R-R clone 10A, suggesting that EGFR inhibition may aid in overcoming resistance (Figure 3D). Similar results were obtained for Cetuximab, a monoclonal antibody that blocks EGFR and is approved for metastatic colorectal cancer (Supplemental Figure 7C).

### PTEN loss alone does not cause PI3K inhibitor resistance

**Figure 4:**
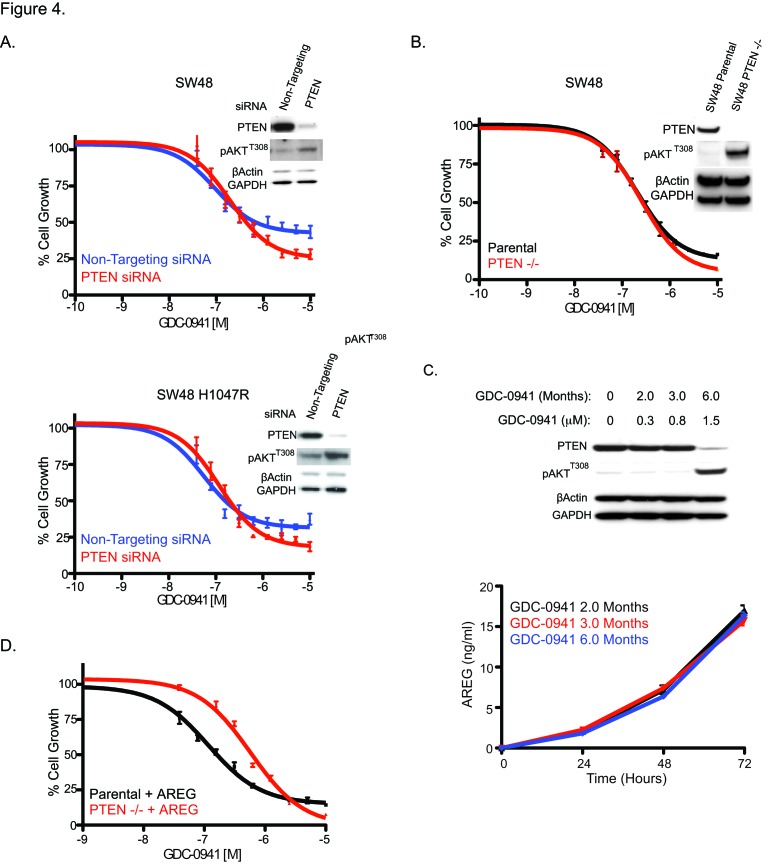
PTEN loss is not responsible for GDC-0941 resistance, but does enhance resistance due to secretion of AREG. (A) SW48 and SW48 H1047R cells transfected with either a non-targeting or PTEN siRNA for 24 hrs before being treated with a dose escalation of GDC-0941. At 72 hrs post dosing cells were assayed for viability and lysates were collected for immunoblotting. (B) SW48 and SW48 PTEN−/− cells treated with a dose escalation of GDC-0941 and assayed for viability 96 hrs after dosing. (C) SW48 cells assessed for PTEN protein expression and pAKT^T308^ levels by immunoblotting after GDC-0941 culture for 2, 3 or 6 months (Top). The same cells assessed for AREG levels by ELISA at 24, 48, and 72 hrs after plating (bottom). (D) SW48 parental and SW48 PTEN−/− cells treated with a dose titration of GDC-0941 with and without 50 ng/ml of AREG and assayed for viability 96 hrs after dosing.

Changes in the production of AREG and PTEN expression were observed in clones made resistant to GDC-0941. To determine how these changes contributed to GDC-0941 resistance, we assessed AREG production and PTEN loss independently. We have already shown that AREG and other EGFR ligands subvert growth inhibition by GDC-0941 in SW48 and SW48 H1047R parental lines (Figure 1B). To investigate the consequence of PTEN loss on SW48 and SW48 H1047R lines, PTEN levels were reduced by siRNA in parental lines and tested for sensitivity to GDC-0941 (Figure 4A). In both cell lines PTEN knockdown failed to change GDC-0941 sensitivity when compared to the same cell lines transfected with a non-targeting control siRNA (Figure 4A). Levels of PTEN knockdown were assessed by western blot 24 hrs after transfection, at the time when cells were dosed with GDC-0941 (Supplemental Figure 6A) and at 72 hrs, the same time viability assays were performed (Figure 4A). To confirm the siRNA data, a matched set of isogenic SW48 cell lines that included parental cells and a clone altered to have deletion of PTEN were assessed for sensitivity to GDC-0941 (Figure 4B). Complete PTEN loss in SW48 lines did not change the response to GDC-0941, which is in agreement with our findings with PTEN siRNA knockdown. (Figure 4B).

While resistant pools were being generated, they were frozen and stored at various times (2, 3, and 6 months). These cells were utilized to establish the time at which PTEN was lost and AREG secretion was initiated (Figure 4C). PTEN loss and increased pAKT^T308^ were not observed until the 6-month time point. However, AREG secretion was observed in resistant pools at 2 months (Figure 4C). PTEN loss did not appear to cause GDC-0941 resistance, but we found that it enhances the resistance generated when SW48 cells are stimulated by EGFR ligands (Figure 4D). When the decrease in GDC-0941 sensitivity in both SW48 cells and SW48 PTEN−/− cells are compared in the presence of AREG, the SW48 PTEN−/− cells are 5-fold more resistant than parental SW48 cells (Figure 4D). Other EGFR ligands, EGF and TGFα were 4.5-fold, and 3-fold, respectively, more resistant in the SW48 PTEN −/− line (Supplemental Figure 6B).

### Resistant clones are sensitive to GDC-0941 in combination with MAPK pathway inhibitors

**Figure 5:**
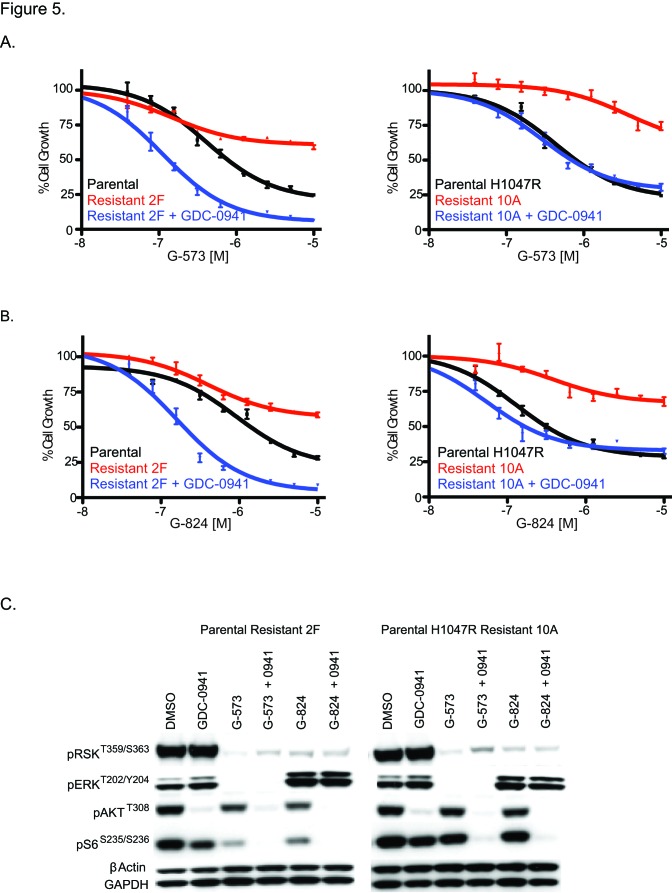
GDC-0941 resistant clones are sensitive to inhibitors of the MAPK pathway in the presence of GDC-0941. (A) SW48 and SW48 H1047R parental and GDC-0941 resistant clones treated with a dose escalation of a MEK inhibitor (G-573) with and without a constant 1.5 uM dose of GDC-0941 and assayed for viability 96 hrs after dosing. (B) SW48 and SW48 H1047R parental and GDC-0941 resistant clones treated with a dose escalation of an ERK inhibitor (G-824) with and without a constant 1.5 uM dose of GDC-0941 and assayed for viability 96 hrs after dosing. (C) SW48 and SW48 H1047R GDC-0941 resistant clones dosed with DMSO, GDC-0941 (1.5 uM), G-573 (1.0 uM), G-824 (1.0 uM) or combinations of these inhibitors at these concentrations. Lysates were collected 24 hrs post treatment. Cell lysates were prepared and analyzed by immunoblotting.

We next wanted to determine how to treat tumor cells once they acquired GDC-0941 resistance. In standard cell culture conditions, we found evidence of MAPK pathway activation (Figure 3C), which suggested they might be sensitive to inhibitors of this pathway. For these studies we utilized an allosteric MEK inhibitor, G-573 [29] and an ERK inhibitor, G-824 [30]. When SW48 and SW48 H1047R GDC-0941 resistant clones were tested for response to these inhibitors all clones tested were highly resistant (Figures 5A and B, Supplemental Figures 7B and 7C). Notably, both G-573 and G-824 were able to suppress MAPK signaling in resistant clones as measured by pERK^T202/Y204^ and pRSK^T359/S363^ (Figure 5C, Supplemental Figure 8). Even when the MAPK pathway was reduced with these treatments, the resistant clones still retained high phosphorylated AKT levels due, in part, to PTEN absence (Figures 2B and 5C, Supplemental Figure 8). When the same GDC-0941 resistant clones were dosed with G-573 or G-824 in media containing a 1.5 uM dose of GDC-0941, sensitivity to both MAPK inhibitors was restored (Figure 5A and B, Supplemental Figure 7B and 7C). For SW48 resistant clones (2F and 2G), sensitivity to both the MEKi and ERKi was greater than in the parental line (Figure 5A and B, Supplemental Figures 7B and 7C). We also found that S6 phosphorylation was only fully repressed when resistant clones were treated with GDC-0941 in combination with G-573 or G-824 (Figure 5C, Supplemental Figure 8).

### Resistant clone is less responsive to GDC-0941 *in vivo*

To confirm our *in vitro* findings, GDC-0941 potency against sensitive (9A) and resistant (10A) SW48 H1047R clones was evaluated in vivo. Both models were dosed daily with 50 mg/kg of GDC-0941 for 17 days and assayed for GDC-0941 resistance (Supplemental Figure 9A and 9B). At 50 mg/kg of GDC-0941 no loss of body weight was observed (Supplemental Figure 9A and 9B). Consistent with *in vitro* results, SW48 H1047R (10A) tumors were more resistant to GDC-0941 when compared to the SW48 H1047R (9A) model, with tumor growth inhibition (TGI) of 6% and 42%, respectively.

We also evaluated PI3K and MAPK pathway markers in 9A and 10A vehicle treated tumors that were collected 1 hr post-final dose. We found that SW48 H1047R (9A) tumors retained PTEN protein expression, while SW48 H1047R (10A) did not have detectable PTEN protein levels (Supplemental Figure 9C). Clone 10A tumors also had substantially elevated AKT phosphorylation compared to clone 9A (Supplemental Figure 9C).

### Resistant clone efficacy can be restored by MAPK inhibition

**Figure 6:**
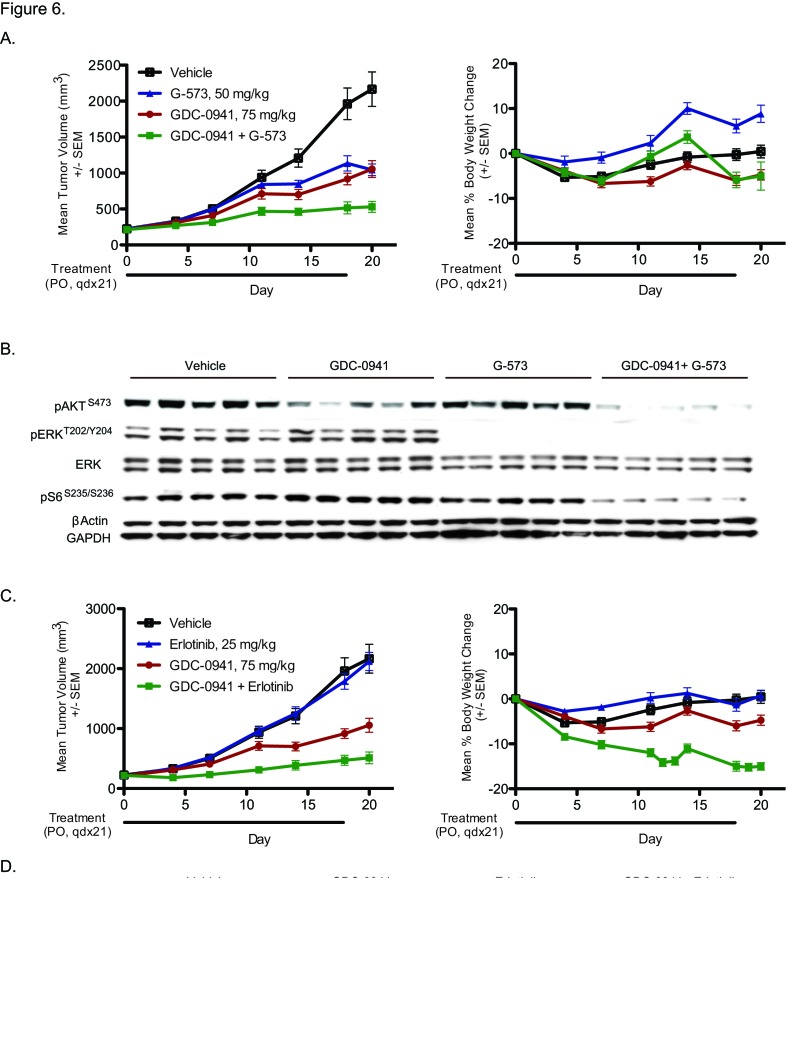
Combination efficacy of PI3K and MEK inhibition in GDC-0941 resistant xenografts. (A) SW48 H1047R resistant (clone 10A) tumor-bearing mice were treated orally and daily with vehicle (0.5% methylcellulose, 0.2% tween-80 + 7.5% captisol), 75 mg/kg GDC-0941, 50 mg/kg G-573, or GDC-0941 and G-573 in combination. Mean tumor volumes (mm^3^) and percent body weight change of SW48 H1047R resistant (clone 10A) tumor-bearing mice measured twice weekly for 21 days. (B) PI3K and MAPK pathway markers were assessed after 21 days of treatment. Five tumors are represented in each group. (C) SW48 H1047R resistant (clone 10A) tumor-bearing mice were treated orally and daily with vehicle (0.5% methylcellulose, 0.2% tween-80 + 7.5% captisol), 75 mg/kg GDC-0941, 25 mg/kg erlotinib, or GDC-0941 and erlotinib in combination. Mean tumor volumes (mm^3^) and percent body weight change of SW48 H1047R resistant (clone 10A) tumor-bearing mice measured twice weekly for 21 days. (D) PI3K and MAPK pathway markers were assessed after 21 days of treatment. Five tumors are represented in each group.

To further confirm our *in vitro* findings, the resistant SW48 H1047R clone (10A) was evaluated in xenografts in combination with a MEK inhibitor, G-573 (Figure 6A). Animals were dosed daily for 21 days with 75 mg/kg of GDC-0941, 50 mg/kg of G-573, or a combination of both drugs. No body weight loss was observed, and the fitted tumor volumes were used to calculate percent tumor growth inhibition (TGI) (Figure 6A). Single agent treatment with GDC-0941 and G-573 showed decreased tumor growth relative to vehicle, with 48% and 33% TGI, respectively. However, consistent with in vitro modeling, the combination showed an increase in efficacy when compared to either single treatment alone, with a TGI of 77% (Figure 6A). Tumors were collected 1 hr after the final dose was administered and assayed for pathway signaling. GDC-0941 treatment decreased levels of phosphorylated AKT; while G-573 treatment decreased phosphorylated ERK. The drugs in combination decreased phosphorylated AKT and ERK, as well as phosphorylated S6 (Figure 6B).

We observed similar efficacy results in clone 10A xenografts treated with the combination of GDC-0941 and erlotinib (Figure 6C). While erlotinib did not show any single agent activity, the GDC-0941 and erlotinib combination resulted in an 89% TGI, which was a substantial increase over single agent GDC-0941 and erlotinib treatment (TGI of 48% and −3%, respectively). Some weight loss was observed with the GDC-0941 and erlotinib combination, however all mice were otherwise healthy and remained on study throughout. A reduction in phosphorylated S6 was also detected in tumors collected 1 hr after the final GDC-0941 and erlotinib combination dose (Figure 6D).

## DISCUSSION

With the emergence of several PI3K inhibitors in clinical trials, it has become increasingly important to study molecular mechanisms that cancer cells may utilize to resist the beneficial effects of these inhibitors [7]. Here we describe the role of EGFR ligands and loss of PTEN in acquired GDC-0941 resistance. By using SW48, SW48 H1047R and a panel of CRC cell lines, we have shown that GDC-0941 growth inhibition can be overcome with the addition of EGFR ligands through MAPK pathway activation. In addition, SW48 and SW48 H1047R cells with acquired resistance to GDC-0941 begin to secrete AREG to bypass suppression of the PI3K pathway. To drive PI3K pathway signaling, resistant cells eventually lose PTEN, which results in increased levels of phosphorylated AKT in the absence of GDC-0941. The loss of PTEN alone is not able to induce resistance to GDC-0941, but it enhances the resistance induced by EGFR stimulation.

AREG has been implicated in resistance to other therapies [31, 32]. Consistent with our findings linking signaling upstream of the PI3K pathway and PI3K inhibitor resistance, other studies have implicated MET in mouse models of acquired resistance and KRAS as a marker for intrinsic resistance [33]. Due to its location downstream of EGFR, the MAPK pathway remains active despite PI3K inhibition. This is consistent with described mechanisms of PI3K inhibitor resistance that demonstrate a reliance on other pathways such as, c-MYC, eIF4E, Notch1, and RSK3/4 [20-22]. It is also worth noting that a gatekeeper resistance mutation has not been described in the PI3K enzymes [34]. In our studies, we sequenced the PIKC3A exons and found no mutations in SW48 sensitive or resistant models.

**Figure 7:**
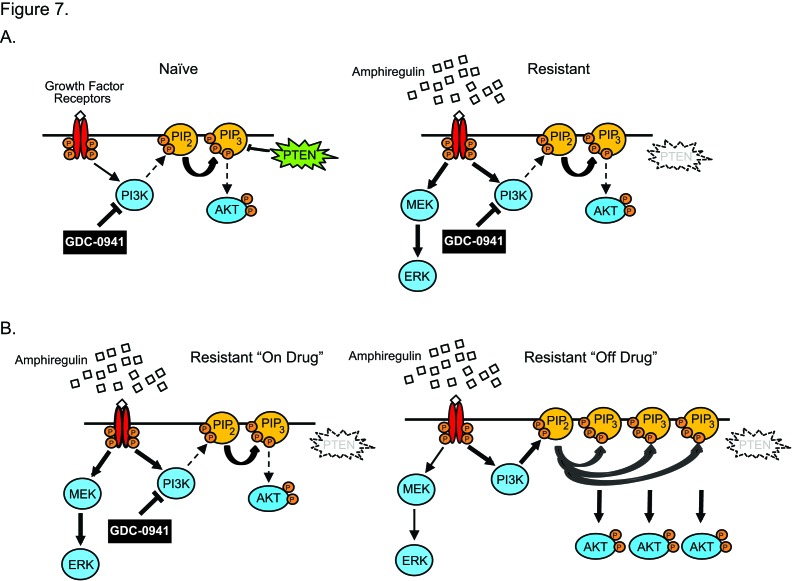
Signaling changes that are observed with GDC-0941 resistance in SW48 cells. (A) SW48 cells are initially sensitive to GDC-0941. GDC-0941 resistance develops with autocrine (AREG/EGFR) signaling and activation of the MAPK pathway. Cells eventually lose PTEN protein as resistance develops. (B) Resistant cells must be maintained on GDC-0941 to prevent signaling flare. In the absence of GDC-0941, resistant cells signal strongly through the PI3K pathway.

The parental SW48 isogenic lines are initially sensitive to GDC-0941. Thus, the cells have reliance on the PI3K pathway that is changed over time in culture in the presence of GDC-0941. MAPK pathway activation through AREG and EGFR allow the cells to grow and survive while the PI3K pathway is blocked by GDC-0941. However, it appears that SW48 cells in culture with GDC-0941 may attempt to maintain signaling through the PI3K pathway through loss of PTEN (Figure 7A). Autocrine signaling through AREG and EGFR activation would activate the PI3K pathway in the absence of GDC-0941. The observed loss of PTEN in resistant clones also supports a mechanism for resistant clones to continue PI3K pathway signaling, especially since PTEN loss did not appear to have a substantial role in GDC-0941 resistance. In the absence of GDC-0941, PTEN null resistant clones continue to secrete AREG and stimulate the EGFR activating the MAPK and PI3K pathways (Figure 7B).

Without a significant role in GDC-0941 resistance, the PTEN loss remains important because it may influence treatment decisions for patients in the clinical setting once resistance occurs. We have shown that once PI3K inhibition is removed, cells that have acquired GDC-0941 resistance also become resistant to MEK or ERK inhibition. The resistance to MAPK pathway inhibition is likely linked to PTEN absence and subsequent hyper-activation of the PI3K/AKT pathway that is observed with the increase in AKT phosphorylation upon release of GDC-0941. The loss of sensitivity to ERK and MEK inhibition can be restored if GDC-0941 is retained in the media, which prevents PI3K/AKT pathway activation. Taken together, these findings may suggest maintenance therapy in the clinic for patients that become resistant to PI3K inhibitors.

## MATERIALS AND METHODS

### Cell culture

Cell lines were cultured McCoy's 5A supplemented with 10% fetal bovine serum, 100 units/ml penicillin, and 100 μg/ml streptomycin at 37°C under 5% CO2. SW48 Parental, SW48 H1047R, and SW48 PTEN (−/−) were licensed from Horizon Discovery Ltd.

### Compounds and reagents

GDC-0941, erlotinib, G-573, and G-824 were all obtained from Genentech. Cetuximab was produced by ImClone Systems, Inc.

### Secreted factor screen

Recombinant purified secreted factors were purchased from Peprotech and R&D Systems and were reconstituted in PBS/0.1% BSA (Supplementary Table 1). Secreted factors were transferred into 96-well plates at a concentration of 1 ug/ml, and subsequently diluted to 100 ng/ml in media containing no drug or 1 uM of GDC-0941. Equal volumes of diluted factor (50 ng/ml final) were arrayed into 384 well plates pre-seeded with cells (2000 cells/well seeded the day before) using an Oasis liquid handler. After 72 hrs, cell viability was determined using CellTiter-Glo (Promega).

### Resistant cell line selection

SW48 and SW48 H1047R cells were grown in increasing concentrations of GDC-0941 until they grew normally in 1.5 umol/L. Resistant clones were isolated by fluorescence-activated cell sorting of individual cells into 96-well plates.

### Western blot and ELISA analyses

Equal amounts of protein were separated by electrophoresis through NuPage Bis-Tris 4-12% gradient gels (Invitrogen), proteins were transferred onto nitrocellulose pore membranes using the iBlot system and protocol from Invitrogen (Carlsbad, CA). PTEN, pAKT (T308), pAkt (Ser473), pERK (T202/Y204), Total EGFR, pEGFR (Y1068), and pS6 (S235/236) antibodies were obtained from Cell Signaling Technology (Danvers, MA). pRSK (T359/S363) antibody was obtained from EMD Millipore (Billerica, Massachusetts). ELISA kits for AREG, βcellulin, HRG-1, EGF, HB-EGF, and TGFα, were obtained from R&D systems (Minneapolis, MN) and carried out according to provided instructions.

### cell viability assays

Cells were seeded (1500 cells/well) in 384-well plates for 16 h. On day two, nine serial 1:2 compound dilutions were made in DMSO in a 96 well plate. The compounds were further diluted into growth media using a Rapidplate robot (Zymark Corp., Hopkinton, MA). The diluted compounds were added to quadruplicate wells in the 384-well cell plate and incubated at 37 C and 5% CO2. After 4 days, relative numbers of viable cells were measured by luminescence using CellTiter-Glo (Promega) according to the manufacturer's instructions and read on a Wallac Multilabel Reader (PerkinElmer, Foster City). EC_50_ values were calculated using Prism 4.0 software (Graphpad, San Diego).

### siRNAs and transfections

PTEN and the non-targeting control siRNAs were from Dharmacon. Transfection of siRNAs was accomplished by Amaxa Nucleofector Kit as described by the manufacturer for solution kit R program T-020.

### In Vivo Methods

All in vivo efficacy and pharmacodynamic studies were reviewed and approved by the Institutional Animal Care and Use Committee (IACUC) at Genentech, and mice were maintained according to the ILAR *Guide for the Care and Use of Laboratory Animals*. Twelve to sixteen week old naïve female C.B-17 SCID beige mice (Charles River Laboratories, San Diego, CA) were inoculated subcutaneously with either 1 million SW48 H1074R clone 9A cells or 5 million SW48 H1074R-R clone 10A cells suspended 1:1 in HBSS:matrigel. Once tumors were established, mice with similarly sized tumors (mean volume of 200-300 mm^3^) were randomized into treatment cohorts. All mice were dosed daily for 17 or 21 days, orally, with vehicle [0.5% methylcellulose/0.2% tween-80 (MCT)], GDC-0941 at 50 or 75 mg/kg, G-573 at 75 mg/kg, or erlotinib at 25 mg/kg. Combination drugs were dosed in succession at the single agent doses indicated. Length (*l*) and width (*w*) of each tumor were measured using digital calipers (Fred V. Fowler Company, Inc., Newton, MA) and tumor volumes were calculated (*V* = *lw*^2^ × 0.5). A linear mixed modeling approach was used to analyze repeated measurement of tumor volumes from the same animals over time. Curve fitting was applied to Log_2_ transformed individual tumor volume data using a linear mixed-effects (LME) model with the R package nlme, version 3.1-97 in R v2.13.0 (R Development Core Team 2008; R Foundation for Statistical Computing; Vienna, Austria). Tumor growth inhibition (%TGI) was calculated as the percentage of the area under the fitted curve (AUC) for the respective dose group/day in relation to the vehicle, such that %TGI = 100 × (1 – (AUC_treatment_/day)/(AUC_vehicle_/day)). Tumor sizes and body weights were recorded twice weekly over the course of the study. Mice with tumor volumes ≥ 2000 mm^3^ or with body weight losses ≥ 20% from their weight at the start of treatment were euthanized per IACUC guidelines.

For pharmacodynamic marker analysis, xenograft tumors (n=5) were excised from animals one hour post-final dose and immediately snap frozen in LN_2_. Frozen tumors were weighed and processed using a pestle (Scienceware; Pequannock, NJ) in 1x Cell Extraction Buffer. Serum samples were also collected from the same five mice per group one hour following the final dose to determine AREG levels.

## SUPPLEMENTAL FIGURES AND TABLES


