# Supplementary material for: Amphiregulin and PTEN evoke a multimodal mechanism of acquired resistance to PI3K inhibition
Source: Genes Cancer. 2014 Mar;5(3-4):113–26. doi: 10.18632/genesandcancer.10 (PMC4091530; doi:10.18632/genesandcancer.10)
Supplement: Supplementary file 1 [file ganc-05-113-s001.pdf]

Amphiregulin and PTEN evoke a multimodal mechanism of acquired resistance to PI3K inhibition

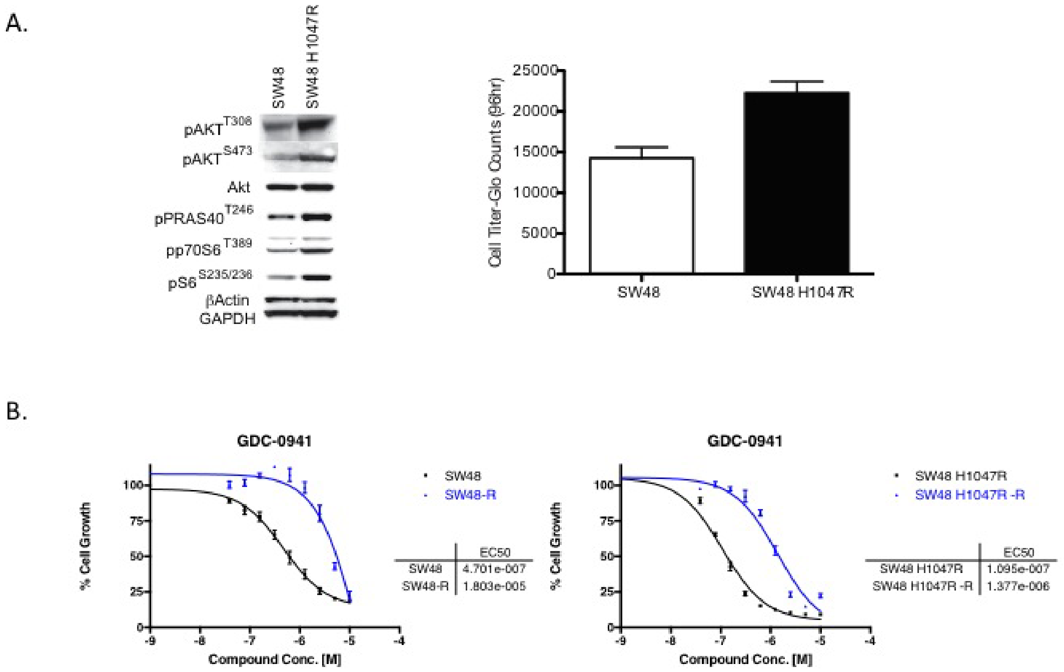

Supplemental Figure 1: A) Characterization of SW48 and SW48 H1047R cell lines. Basal levels of PI3K pathway members assessed by immunoblotting in both SW48 and SW48 with a knock-in H1047R PI3Kα mutation (left). Cell growth of the two cell lines in culture. Equal numbers of cells were plated and the total number of cells after 4 days was determined by CellTiter-Glo (right). B) Characterization of SW48 GDC-0941 resistant pools. SW48 and SW48 H1047R GDC-0941 parental and resistant pools treated with a dose escalation of GDC-0941 and assayed for viability using CellTiter-Glo 96 hrs post dosing.

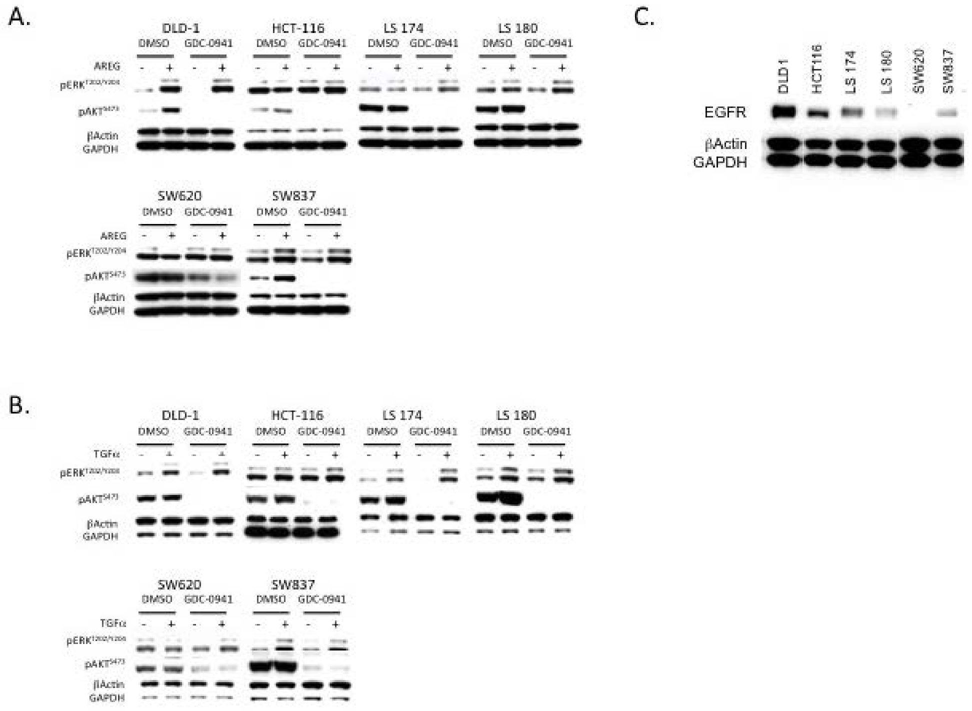

Supplemental Figure 2: A) Amphiregulin (AREG) stimulated signaling in a CRC cell line panel. Cells were stimulated with 50 ng/ml of AREG for one hr in the presence or absence of GDC-0941. B) TGFα stimulated signaling in a CRC cell line panel. Cells were stimulated with 50 ng/ml of TGFα for one hr in the presence or absence of GDC-0941. C) EGFR protein levels in CRC cell line panel.

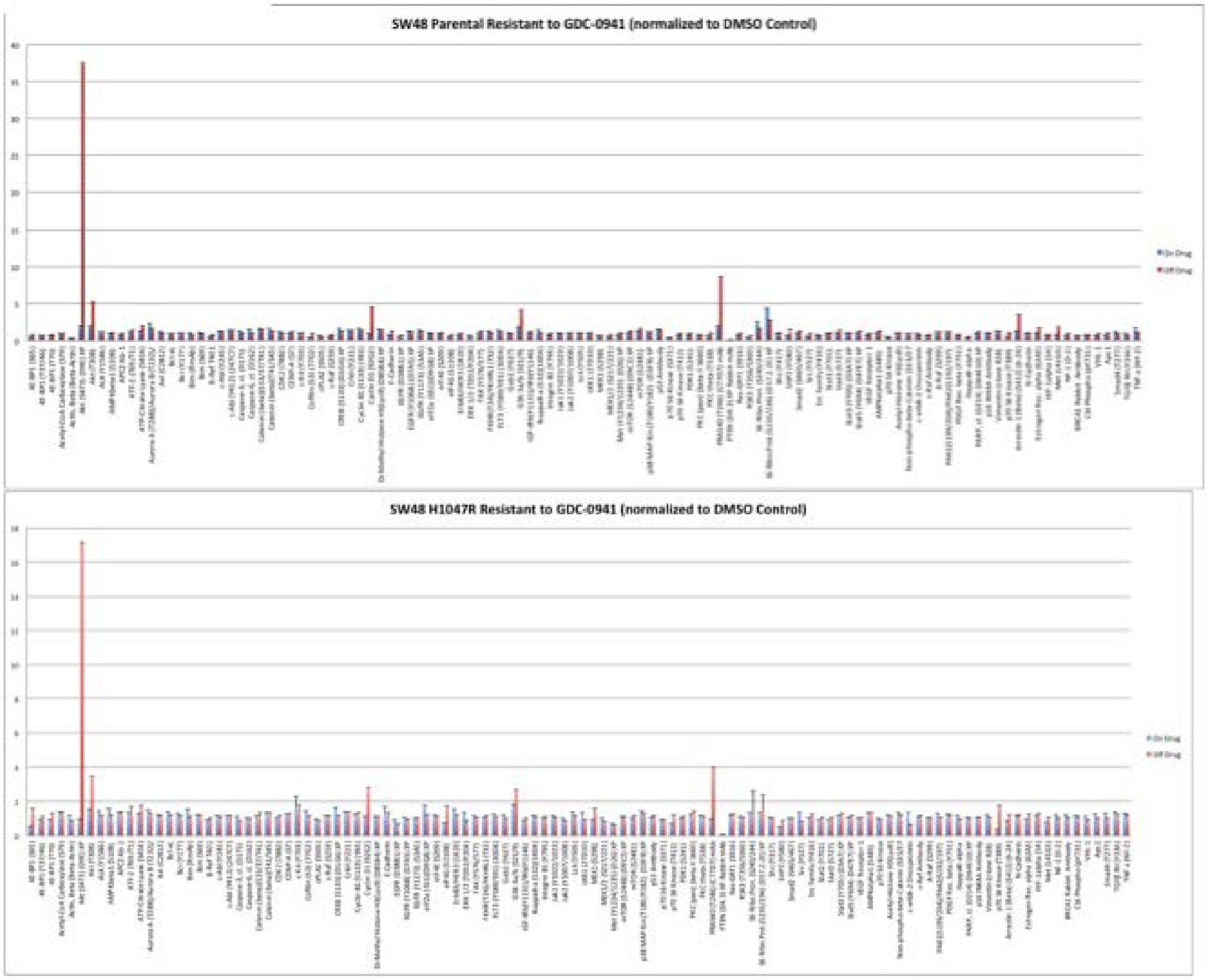

Supplemental Figure 3: Characterization of SW48 GDC-0941 resistant pools by Reverse Phase Protein Array (RPPA). Protein levels of SW48 and SW48 H1047R GDC-0941 resistant pools treated with DMSO or 1.5 μM of GDC-0941 for 48hrs normalized to protein levels in the respective parental line treated with DMSO for 48hrs.

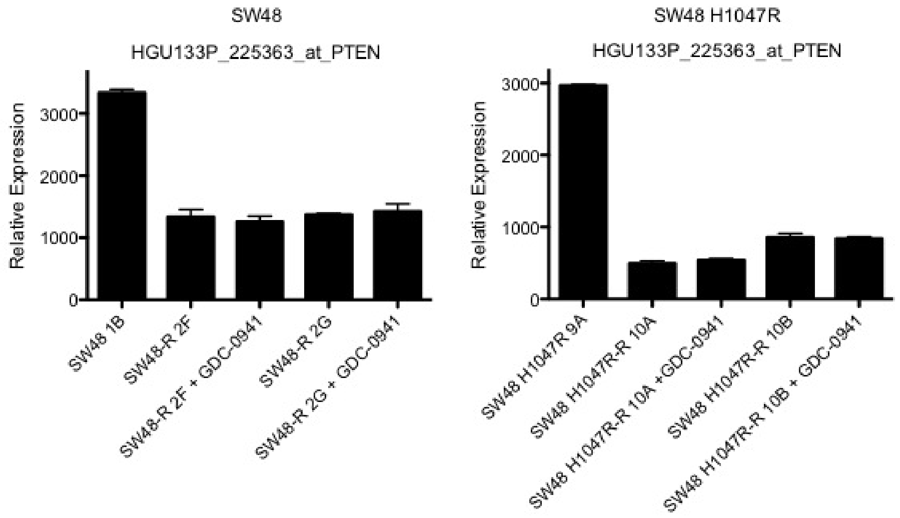

Supplemental Figure 4: PTEN transcript levels in SW48 GDC-0941 resistant clones. PTEN mRNA levels of SW48 and SW48 H1047R GDC-0941 resistant clones treated with DMSO or 1.5 μM of GDC-0941 for 48hrs.

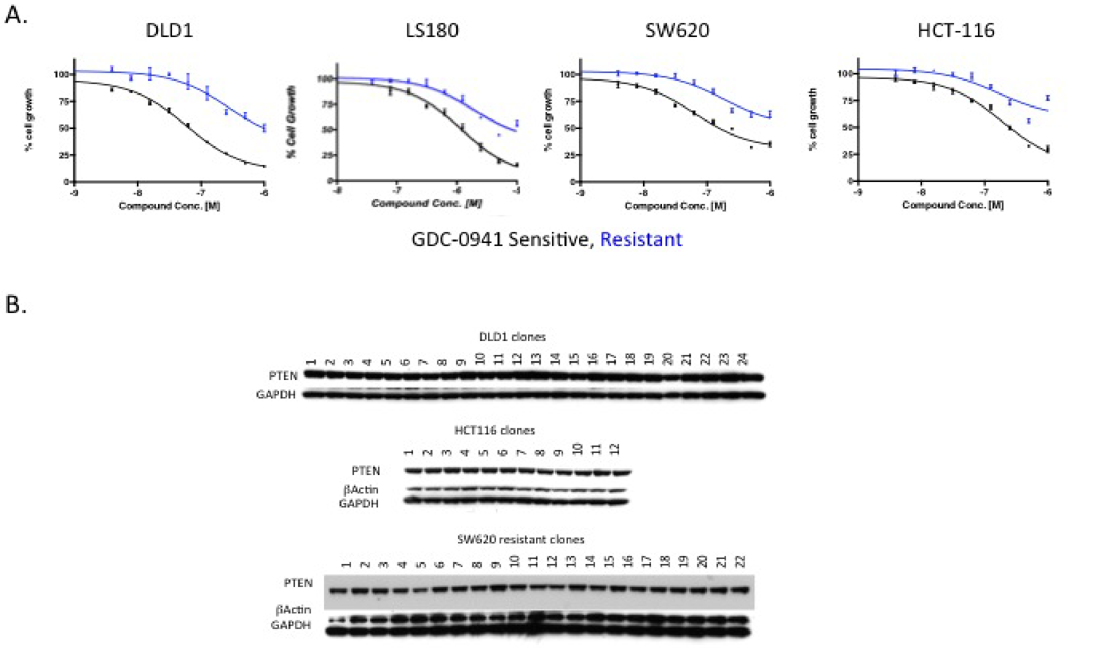

Supplemental Figure 5: A) Characterization of additional GDC-0941 resistant colorectal cancer cell lines. DLD1, DLD1-R, HCT-116, HCT-116-R, LS-180, LS-180-R, SW620, and SW620-R were assayed for viability using CellTiter-Glo 96 hrs post dosing. B) PTEN protein levels in additional GDC-0941 resistant colorectal cancer cell lines. Clones generated from DLD1-R, HCT-116-R, and SW620-R cells were collected for immunoblotting and probed for PTEN protein levels.

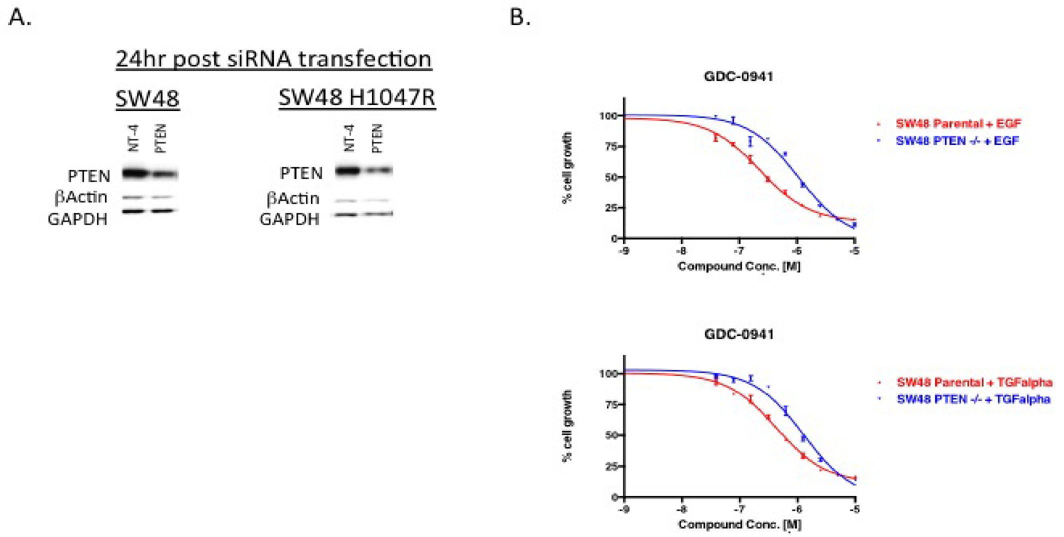

Supplemental Figure 6: A) SW48 and SW48 H1047R cells transfected with either a non-targeting or PTEN siRNA for 24 hrs before being collected for immunoblotting to be probed for PTEN protein levels. B) SW48 parental and SW48 PTEN-/- cells treated with a dose titration of GDC-0941 with and without 50 ng/ml of EGF or TGFα and assayed for viability using CellTiter-Glo 96 hrs after dosing.

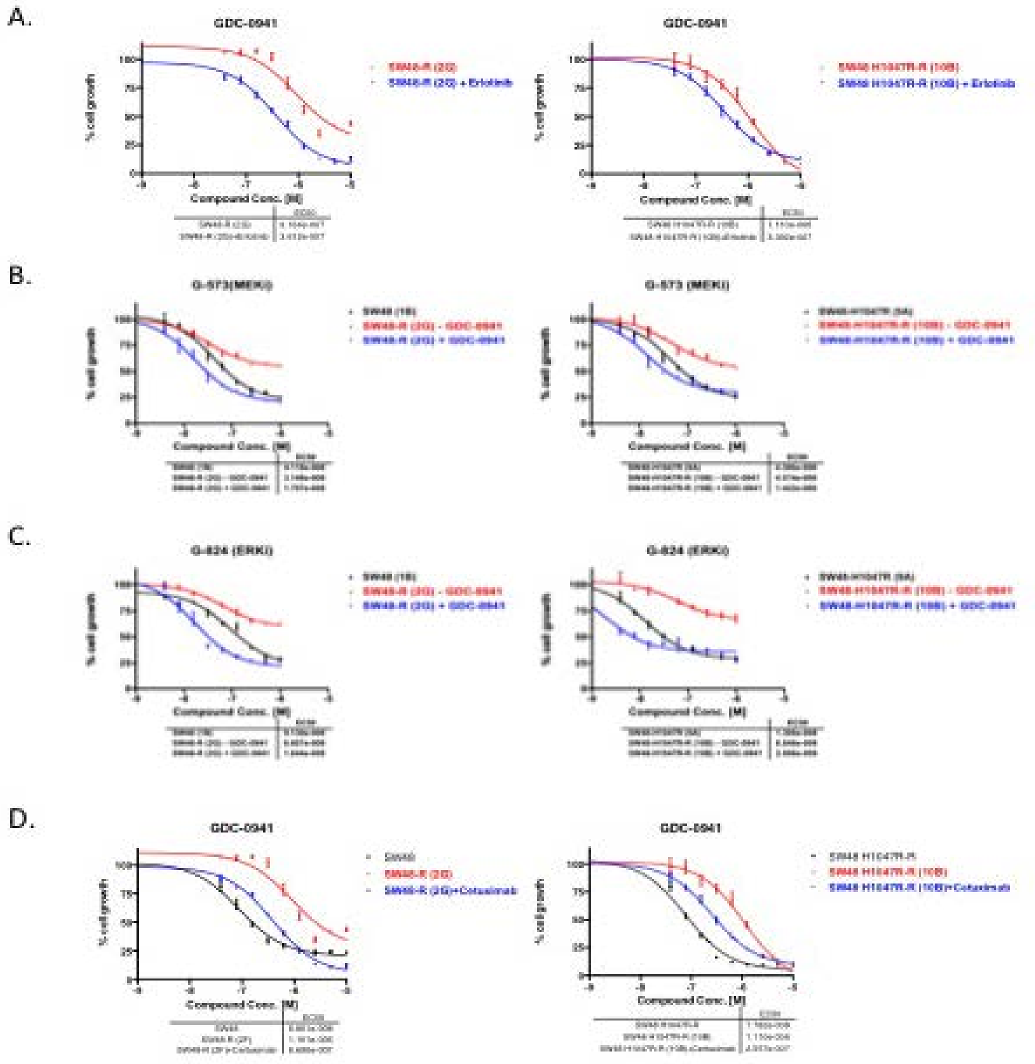

Supplemental Figure 7: A) SW48 and SW48 H1047R GDC-0941 resistant clones were treated with a dose escalation of GDC-0941 with and without a constant 1 μM dose of erlotinib and assayed for viability using CellTiter-Glo 96 hrs post dosing. B) GDC-0941 resistant clones are sensitive to inhibitors of the MAPK pathway in the presence of GDC-0941. SW48 and SW48 H1047R parental and GDC-0941 resistant clones treated with a dose escalation of a MEK inhibitor (G-573) with and without a constant 1.5 μM dose of GDC-0941 and assayed for viability using CellTiter-Glo 96 hrs after dosing. C) GDC-0941 resistant clones are sensitive to inhibitors of the MAPK pathway in the presence of GDC-0941. SW48 and SW48 H1047R parental and GDC-0941 resistant clones treated with a dose escalation of an ERK inhibitor (G-824) with and without a constant 1.5 μM dose of GDC-0941 and assayed for viability using CellTiter-Glo 96 hrs after dosing. D) GDC-0941 resistant clones are sensitive to EGFR inhibition by Cetuximab in the presence of GDC-0941. SW48 and SW48 H1047R parental and GDC-0941 resistant clones treated with a dose escalation of an Cetuximab with and without a constant 1.5 μM dose of GDC-0941 and assayed for viability using CellTiter-Glo 96 hrs after dosing.

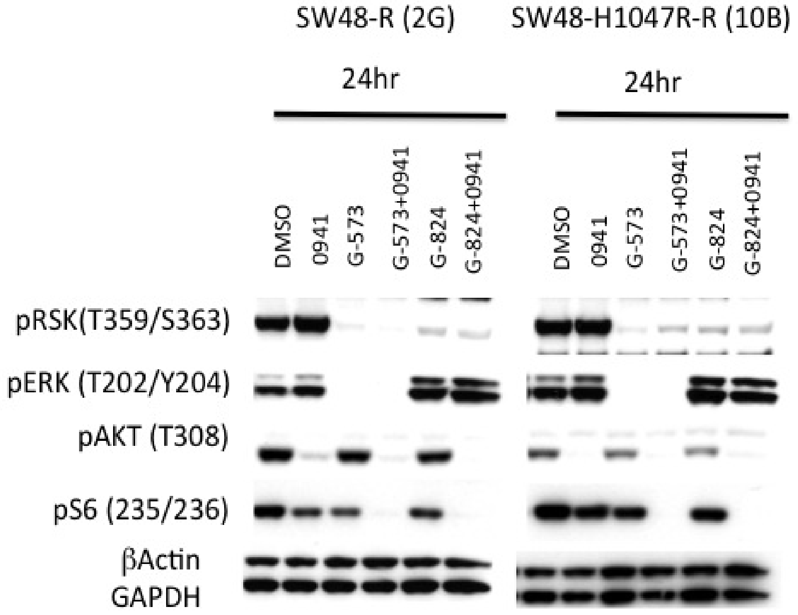

Supplemental Figure 8: GDC-0941 resistant clones are sensitive to inhibitors of the MAPK pathway in the presence of GDC-0941. SW48 and SW48 H1047R GDC-0941 resistant clones dosed with DMSO, GDC-0941 (1.5 μM), G-573 (1.0 μM), G-824 (1.0 μM) or combinations of these inhibitors at these concentrations. Lysates were collected 24 hrs post treatment. Cell lysates were prepared and analyzed by immunoblotting.

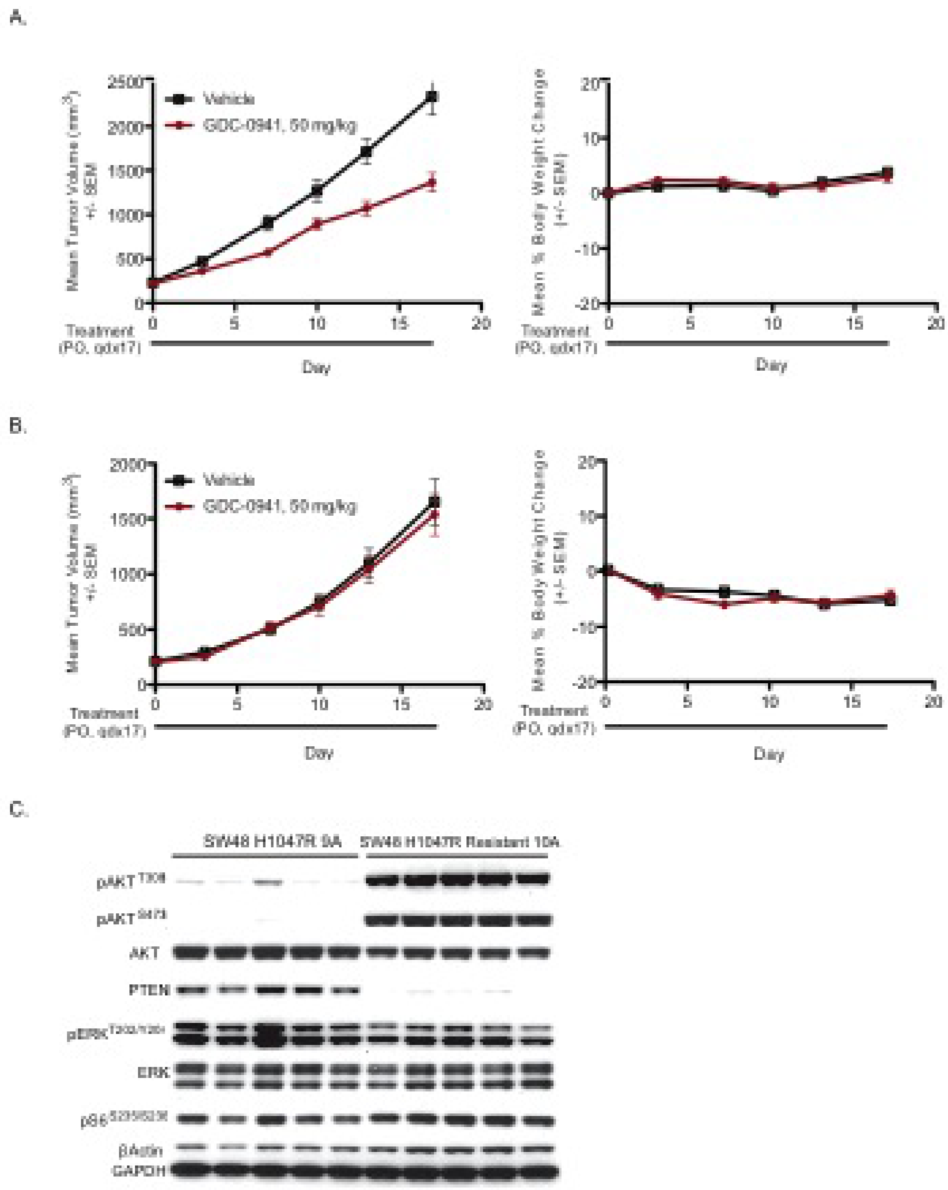

Supplemental Figure 9. Single agent potency of GDC-0941 in SW48 H1047R parental and resistant clone xenografts. (A) SW48 H1047R sensitive (clone 9A) and (B) resistant (clone 10A) tumor-bearing mice were treated orally and daily with vehicle (0.5% methylcellulose, 0.2% tween-80) or 50 mg/kg GDC-0941 for 17days. Mean percent body weight change (%) is also shown. (C) Resistant tumors have increased PI3K pathway signaling in vivo. Immunoblots from SW48 H1047R sensitive (clone 9A) and resistant (clone 10A) vehicle treated tumor-bearing mice. Five tumors are represented in each group.

Supplemental Table 1: List of 418 factors screened to investigate the potential role of soluble ligands in resistance to GDC-0941.

Catalog Number	Product name	Company
310-11	4-1BB Ligand	Peprotech
310-15	4-1BB Receptor	Peprotech
120-14	Activin-A (Insect cell derived)	Peprotech
120-15	Activin-B (Insect cell derived)	Peprotech
310-22	AITRL	Peprotech
100-55B	Amphiregulin (98 a.a.)	Peprotech
130-06	ANG-1 (HeLa cell derived)	Peprotech
130-07	ANG-2 (CHO cell derived)	Peprotech
310-10C	APRIL (insect cell derived)	Peprotech
450-17	Artemin	Peprotech
310-13	BAFF	Peprotech
310-13R	BAFF Receptor	Peprotech
300-47	BCA-1/BLC (CXCL13)	Peprotech
310-16	BCMA	Peprotech
450-02	BDNF	Peprotech
100-50	Betacellulin	Peprotech
450-01	beta-NGF	Peprotech
1927-ZN-010	BMP-1/PCP	R&D
2926-BP-025	BMP-10	R&D
120-04	BMP-13/CDMP-2	Peprotech
120-02	BMP-2	Peprotech
120-24B	BMP-3	Peprotech
120-05	BMP-4 (HeLa cell derived)	Peprotech
120-05ET	BMP-4 (truncated; E.coli derived)	Peprotech
615-BMC-020	BMP-5	R&D
120-06	BMP-6 (HEK 293 cell derived)	Peprotech
120-03	BMP-7 (CHO cell derived)	Peprotech
1073-BP-010	BMP-8a	R&D
3209-BP-010	BMP-9	R&D
2036-BC-050	BOC	R&D
300-50	BRAK (CXCL14)	Peprotech
300-32	Cardiotrophin-1	Peprotech
272-I-010	CCL1/I-309/TCA-3	R&D
320-EO-020	CCL11/Eotaxin	R&D
327-P4-025	CCL13/MCP-4	R&D
324-HC-010	CCL14a/HCC-1	R&D
363-MG-025	CCL15/MIP-1 delta	R&D
628-LK-025	CCL15/MIP-1 delta	R&D
802-HC-025	CCL16/HCC-4	R&D
364-DN-025	CCL17/TARC	R&D
394-PA-010	CCL18/PARC	R&D
361-MI-025	CCL19/MIP-3 beta	R&D

279-MC-010	CCL2/JE/MCP-1	R&D
360-MP-025	CCL20/MIP-3 alpha	R&D
366-6C-025	CCL21/6Ckine	R&D
336-MD-025	CCL22/MDC	R&D
508-CK-025	CCL23/Ck beta 8-1	R&D
371-MP-025	CCL23/MPIF-1	R&D
343-E2-025	CCL24/Eotaxin-2/MPIF-2	R&D
334-TK-025	CCL25/TECK	R&D
653-E3-025	CCL26/Eotaxin-3	R&D
376-CT-025	CCL27/CTACK	R&D
717-VC-025	CCL28	R&D
270-LD-010	CCL3/MIP-1 alpha	R&D
509-MI-025	CCL3L1/MIP-1 alpha Isoform LD78 be	R&D
271-BME-010	CCL4/MIP-1 beta	R&D
3046-MB-025	CCL4L1/LAG-1	R&D
278-RN-010	CCL5/RANTES	R&D
282-P3-010	CCL7/MCP-3/MARC	R&D
281-CP-010	CCL8/MCP-2	R&D
100-01	CD22 (CHO cell derived)	Peprotech
1028-CL-050	CD30 Ligand/TNFSF8	R&D
6420-CL-025	CD40 Ligand/TNFSF5	R&D
450-05	CDNF	Peprotech
300-66	Chemerin	Peprotech
450-13	CNTF	Peprotech
300-54	CTACK (CXL27)	Peprotech
120-19	CTGF	Peprotech
120-16	CTGFL/WISP-2	Peprotech
365-FR-025	CX3CL1/Fractalkine	R&D
275-GR-010	CXCL1/GRO alpha/KC/CINC-1	R&D
266-IP-010	CXCL10/IP-10/CRG-2	R&D
672-IT-025	CXCL11/I-TAC	R&D
6448-SD-025	CXCL12/SDF-1	R&D
350-NS-010	CXCL12/SDF-1 alpha	R&D
351-FS-010	CXCL12/SDF-1 beta	R&D
801-CX-025	CXCL13/BLC/BCA-1	R&D
866-CX-025	CXCL14/BRAK	R&D
300-55	CXCL16	Peprotech
4207-DM-025	CXCL17/VCC-1	R&D
276-GB-010	CXCL2/GRO beta/MIP-2/CINC-3	R&D
277-GG-010	CXCL3/GRO gamma/CINC-2/DCIP-1	R&D
795-P4-025	CXCL4/PF4	R&D
649-EN-025	CXCL5/ENA-70	R&D
651-NA-025	CXCL5/ENA-74	R&D
254-XB-025	CXCL5/ENA-78	R&D
333-GC-025	CXCL6/GCP-2	R&D
393-NP-010	CXCL7/NAP-2	R&D
208-IL-010	CXCL8/IL-8	R&D

618-IL-010	CXCL8/IL-8	R&D
392-MG-010	CXCL9/MIG	R&D
120-25	CYR61	Peprotech
4777-DH-050	Desert Hedgehog/Dhh	R&D
120-30	DKK-1 (HEK293 cells)	Peprotech
6628-DK-010	Dkk-2	R&D
1118-DK-050	Dkk-3	R&D
1269-DK-010	Dkk-4	R&D
6148-DR-025	Draxin	R&D
236-EG-01M	EGF	R&D
100-15R	EGF Receptor (CHO cell derived)	Peprotech
100-44	EG-VEGF	Peprotech
300-22	ENA-78/CXCL5 (5-78 a.a.)	Peprotech
300-22B	ENA-78/CXCL5 (8-78 a.a.)	Peprotech
1810-EC-050	Endocan/ESM-1	R&D
150-01	Endostatin	Peprotech
300-21	Eotaxin (CCL11)	Peprotech
300-33	Eotaxin-2 (CCL24)	Peprotech
300-48	Eotaxin-3 (CCL26)	Peprotech
6417-A1-050	Ephrin-A1	R&D
BT359	Ephrin-A3	R&D
359-EA-200	Ephrin-A3	R&D
BT369	Ephrin-A4	R&D
369-EA-200	Ephrin-A4	R&D
BT374	Ephrin-A5	R&D
374-EA-200	Ephrin-A5	R&D
BT395	Ephrin-B3	R&D
395-EB-200	Ephrin-B3	R&D
100-51	Epigen	Peprotech
100-04	Epiregulin	Peprotech
150-15	E-Selectin (CHO cell derived)	Peprotech
300-35	Exodus-2 (CCL21)	Peprotech
126-FL-010	Fas Ligand/TNFSF6	R&D
140-13	Fetuin A/AHSG (HEK293 cell derived)	Peprotech
231-BC-025	FGF acidic	R&D
234-FSE-025	FGF basic	R&D
100-26	FGF-10	Peprotech
2246-FG-025	FGF-12	R&D
100-29	FGF-16	Peprotech
100-27	FGF-17	Peprotech
100-28	FGF-18	Peprotech
100-32	FGF-19	Peprotech
100-41	FGF-20	Peprotech
100-42	FGF-21	Peprotech
3867-FG-025	FGF-22	R&D
100-52	FGF-23	Peprotech
1206-F3-025	FGF-3	R&D

100-31	FGF-4	Peprotech
100-34	FGF-5	Peprotech
100-30	FGF-6	Peprotech
100-25	FGF-8	Peprotech
100-23	FGF-9	Peprotech
100-17A	FGF-acidic	Peprotech
100-18B	FGF-basic	Peprotech
1593-FB-025	FGF-BP	R&D
3095-FB-025	Fibulin 5/DANCE	R&D
308-FK-005	Flt-3 Ligand	R&D
300-19	Flt3-Ligand	Peprotech
120-13	Follistatin	Peprotech
300-31	Fractalkine (CX3CL1)	Peprotech
5988-FZ-050	Frizzled-1	R&D
3459-FZ-050	Frizzled-10	R&D
5847-FZ-050	Frizzled-4	R&D
1617-FZ-050	Frizzled-5	R&D
1617-FZC-050	Frizzled-5	R&D
6178-FZ-050	Frizzled-7	R&D
6129-FZ-050	Frizzled-8	R&D
450-21	gAcrp30/Adipolean	Peprotech
450-20	gAcrp30/Adipolean Variant	Peprotech
450-39	Galectin-1	Peprotech
450-38	Galectin-3	Peprotech
300-41	GCP-2 (CXCL6)	Peprotech
300-23	G-CSF	Peprotech
6937-GD-010	GDF-1	R&D
120-11	GDF-11	Peprotech
120-28	GDF-15/MIC-1 (cell culture derived)	Peprotech
120-07	GDF-2 (CHO cell derived)	Peprotech
120-22	GDF-3	Peprotech
120-01	GDF-5 (BMP-14/CDMP-1)	Peprotech
120-37	GDF-7	Peprotech
788-G8-010	GDF-8/Myostatin	R&D
450-10	GDNF	Peprotech
670-FR-100	GFR alpha-3/GDNF R alpha-3	R&D
694-GL-025	GITR Ligand/TNFSF18	R&D
300-03	GM-CSF	Peprotech
450-37	GMF-beta	Peprotech
5190-GR-050	Gremlin	R&D
300-11	GRO/MGSA (CXCL1)	Peprotech
300-39	GRO-beta (CXCL2)	Peprotech
300-40	GRO-gamma (CXCL3)	Peprotech
100-47	HB-EGF	Peprotech
300-38B	HCC-1/CCL14 (66 a.a.)	Peprotech
300-38	HCC-1/CCL14 (72 a.a.)	Peprotech
100-03	Heregulin-beta1	Peprotech

100-39	HGF (Insect cell derived)	Peprotech
310-27	HVEM-Fc (Insect cell derived)	Peprotech
300-37	I-309 (CCL1)	Peprotech
150-05	ICAM-1 (CHO cell derived)	Peprotech
11350-1	IFN	R&D
11200-1	IFN-alpha	R&D
11175-1	IFN-alpha 1	R&D
11105-1	IFN-alpha 2	R&D
11177-1	IFN-alpha 4	R&D
11180-1	IFN-alpha 4	R&D
11100-1	IFN-alpha A	R&D
11115-1	IFN-alpha B2	R&D
11120-1	IFN-alpha C	R&D
11125-1	IFN-alpha D	R&D
11130-1	IFN-alpha F	R&D
11135-1	IFN-alpha G	R&D
11145-1	IFN-alpha H2	R&D
11150-1	IFN-alpha I	R&D
11160-1	IFN-alpha J1	R&D
11165-1	IFN-alpha K	R&D
11190-1	IFN-alpha WA	R&D
300-02BC	IFN-beta (CHO cell derived)	Peprotech
300-02	IFN-gamma	Peprotech
300-02L	IFN-lambda 1	Peprotech
300-02K	IFN-lambda 2	Peprotech
300-02J	IFN-omega	Peprotech
350-10	IGF-BP1	Peprotech
350-06B	IGF-BP2 (insect cell derived)	Peprotech
100-08	IGF-BP3	Peprotech
350-05B	IGF-BP4 (insect cell derived)	Peprotech
100-05	IGF-BP5	Peprotech
350-07B	IGF-BP6 (insect cell derived)	Peprotech
350-09	IGF-BP7	Peprotech
100-11	IGF-I	Peprotech
100-12	IGF-II	Peprotech
200-LA-010	IL-1 alpha/IL-1F1	R&D
201-LB-005	IL-1 beta/IL-1F2	R&D
200-10	IL-10	Peprotech
200-11	IL-11	Peprotech
219-IL-005	IL-12	R&D
200-12	IL-12p70 (CHO cell derived)	Peprotech
200-12p80H	IL-12p80 (insect cell derived)	Peprotech
200-13	IL-13	Peprotech
200-13A	IL-13 Variant	Peprotech
200-15	IL-15	Peprotech
200-16A	IL-16 (121 a.a.)	Peprotech
200-16	IL-16 (130 a.a.)	Peprotech

317-ILB-050	IL-17/IL-17A	R&D
200-17	IL-17A	Peprotech
200-28	IL-17B	Peprotech
1234-IL-025	IL-17C	R&D
200-27	IL-17D	Peprotech
200-24	IL-17E	Peprotech
200-25	IL-17F	Peprotech
B001-5	IL-18/IL-1F4	R&D
200-19	IL-19	Peprotech
200-01A	IL-1alpha	Peprotech
200-01B	IL-1beta	Peprotech
1975-IL-025	IL-1F7/FIL1 zeta	R&D
200-01RA	IL-1RA	Peprotech
200-02	IL-2	Peprotech
200-20	IL-20	Peprotech
200-21	IL-21	Peprotech
200-22	IL-22	Peprotech
200-23	IL-23 (insect cell derived)	Peprotech
1965-IL-025	IL-24	R&D
1870-IL-010	IL-26/AK155	R&D
1375-IL-025	IL-26/AK155	R&D
2526-IL-010	IL-27	R&D
1587-IL-025	IL-28A/IFN-lambda 2	R&D
5259-IL-025	IL-28B/IFN-lambda 3	R&D
1598-IL-025	IL-29/IFN-lambda 1	R&D
200-03	IL-3	Peprotech
200-31	IL-31	Peprotech
3040-IL-050	IL-32 alpha	R&D
200-33	IL-33	Peprotech
200-34	IL-34 (HEK293 cells)	Peprotech
1078-IL-025	IL-36 alpha/IL-1F6	R&D
1099-IL-025	IL-36 beta/IL-1F8	R&D
2320-IL-025	IL-36 gamma/IL-1F9	R&D
200-04	IL-4	Peprotech
200-05	IL-5	Peprotech
200-06	IL-6	Peprotech
200-07	IL-7	Peprotech
200-08M	IL-8 (72 a.a.) (CXCL8)	Peprotech
200-08	IL-8 (77 a.a.) (CXCL8)	Peprotech
200-09	IL-9	Peprotech
1705-HH-025	Indian Hedgehog/Ihh	R&D
300-12	IP-10 (CXCL10)	Peprotech
300-46	I-TAC (CXCL11)	Peprotech
100-19	KGF (FGF-7)	Peprotech
100-53	KLOTHO (CHO cell derived)	Peprotech
5889-KB-050	Klotho beta	R&D
300-58	LAG-1 (CCL4L1)	Peprotech

300-56	LD78-beta (CCL3L1)	Peprotech
300-44	LEC/NCC-4 (CCL-16)	Peprotech
3468-LE-050	LEDGF	R&D
746-LF-025	Lefty-A	R&D
300-27	Leptin	Peprotech
310-09B	LIGHT (Insect cell derived)	Peprotech
300-20	Lymphotactin (XCL1)	Peprotech
450-06	MANF	Peprotech
130-12	Maspin	Peprotech
300-04	MCP-1/MCAF (CCL2)	Peprotech
300-15	MCP-2 (CCL8)	Peprotech
300-17	MCP-3 (CCL7)	Peprotech
300-24	MCP-4 (CCL13)	Peprotech
300-25	M-CSF	Peprotech
300-36	MDC (67 a.a.) (CCL22)	Peprotech
300-36A	MDC (69 a.a.) (CCL22)	Peprotech
300-57	MEC (CCL28)	Peprotech
130-01	MIA	Peprotech
130-02	MIA-2	Peprotech
450-16	Midkine	Peprotech
300-26	MIG (CXCL9)	Peprotech
300-08	MIP-1alpha (CCL3)	Peprotech
300-09	MIP-1beta (CCL4)	Peprotech
300-29	MIP-3 (CCL23)	Peprotech
300-29A	MIP-3alpha (CCL20)	Peprotech
300-29B	MIP-3beta (CCL19)	Peprotech
300-34	MIP-4 (CCL18)	Peprotech
300-43	MIP-5 (CCL15)	Peprotech
1737-MS-010	MIS/AMH	R&D
120-00	Myostatin	Peprotech
120-12	Myostatin Propeptide	Peprotech
300-14	NAP-2 (CXCL7)	Peprotech
296-HR-050	Neuregulin-1 alpha/NRG1 alpha	R&D
377-HB-050	Neuregulin-1 beta 1/NRG1 beta 1	R&D
378-SM-025	Neuregulin-1 Isoform SMDF	R&D
5898-NR-050	Neuregulin-1/NRG1	R&D
450-36D	Neuritin	Peprotech
3870-N1-025	Neuropilin-1	R&D
2215-N2-025	Neuropilin-2	R&D
450-11	Neurturin	Peprotech
450-18	NNT-1/BCSF-3	Peprotech
3218-ND-025	Nodal	R&D
120-10C	NOGGIN (293 cell derived)	Peprotech
120-26	NOV	Peprotech
450-03	NT-3	Peprotech
450-04	NT-4	Peprotech
295-OM-010	Oncostatin M/OSM	R&D

300-10T	Oncostatin-M (209 a.a.)	Peprotech
300-10	Oncostatin-M (227 a.a.)	Peprotech
450-14	Osteoprotegerin	Peprotech
185-OS-025	Osteoprotegerin/TNFRSF11B	R&D
130-03	OTOR	Peprotech
310-28	OX40 Ligand (Insect cell derived)	Peprotech
120-HD-001	PDGF	R&D
100-13A	PDGF-AA	Peprotech
100-00AB	PDGF-AB	Peprotech
100-14B	PDGF-BB	Peprotech
100-00CC	PDGF-CC	Peprotech
1159-SB-025	PDGF-DD	R&D
150-06	PECAM-1 (HEK293 cell derived)	Peprotech
130-13	PEDF	Peprotech
1826-TS-025	Pentraxin 3/TSG-14	R&D
450-12	Persephin	Peprotech
300-16	PF-4 (CXCL4)	Peprotech
450-15	Pleiotrophin	Peprotech
100-06	PlGF-1	Peprotech
100-56	PlGF-2	Peprotech
100-57	PlGF-3	Peprotech
2420-PG-050	Progranulin/PGRN	R&D
100-46	Prokineticin-2	Peprotech
100-07	Prolactin	Peprotech
100-09	PTHrP	Peprotech
683-RK-100	RANK/TNFRSF11A	R&D
300-06	RANTES (CCL5)	Peprotech
130-15	Relaxin-2	Peprotech
130-10	Relaxin-3	Peprotech
450-22	RELM-beta	Peprotech
450-19	Resistin	Peprotech
4645-RS-025	R-Spondin 1	R&D
3266-RS-025	R-Spondin 2	R&D
3500-RS-025	R-Spondin 3	R&D
4575-RS-025	R-Spondin 4	R&D
310-29	sCD100 (CHO cell derived)	Peprotech
110-01	sCD14 (293 cell derived)	Peprotech
310-26	sCD23	Peprotech
310-30	sCD27 Ligand (CHO cell derived)	Peprotech
450-42	sCD30 Ligand (CHO cell derived)	Peprotech
310-31	sCD34 (CHO cell derived)	Peprotech
310-02	sCD40 Ligand	Peprotech
300-07	SCF	Peprotech
100-22A	SCGF-alpha	Peprotech
100-22B	SCGF-beta	Peprotech
300-28A	SDF-1alpha (CXCL12)	Peprotech
300-28B	SDF-1beta (CXCL12)	Peprotech

140-08	sDLL-1 (HEK293 cell derived)	Peprotech
140-07	sDLL-4 (HEK293 cell derived)	Peprotech
310-03H	sFas Ligand (CHO cell derived)	Peprotech
310-20	sFas Receptor	Peprotech
120-29	sFRP-1 (HeLa cell derived)	Peprotech
192-SF-010	sFRP-3	R&D
1827-SF-025	sFRP-4	R&D
6266-SF-050	sFRP-5	R&D
200-02R	sIL-2Ralpha (Insect cell derived)	Peprotech
200-04R	sIL-4Ralpha (HEK293 cell derived)	Peprotech
200-06R	sIL-6Ralpha (HEK293 cell derived)	Peprotech
150-11	Slit2-N (HEK293 cell derived)	Peprotech
1549-S1-025	Soggy-1/DkkL1	R&D
100-45	Sonic Hedgehog	Peprotech
1406-ST-025	SOST/Sclerostin	R&D
120-36	SPARC/Osteonectin (CHO cell derivec	Peprotech
310-01	sRANK Ligand	Peprotech
310-08	sRANK Receptor	Peprotech
310-07	sTNF-receptor Type I	Peprotech
310-12	sTNF-receptor Type II	Peprotech
310-18	sTRAIL receptor-1	Peprotech
310-19	sTRAIL receptor-2	Peprotech
310-04	sTRAIL/Apo2L	Peprotech
310-17	TACI	Peprotech
5154-TA-050	TAFA1/FAM19A1	R&D
300-63	TAFA-2	Peprotech
4179-TA-050	TAFA2/FAM19A2	R&D
5099-TA-050	TAFA4/FAM19A4	R&D
5148-TA-050	TAFA5/FAM19A5	R&D
300-30	TARC (CCL17)	Peprotech
300-45	TECK (CCL25)	Peprotech
300-60	TFF-1	Peprotech
300-59	TFF-2	Peprotech
300-61	TFF-3	Peprotech
100-16A	TGF-alpha	Peprotech
240-B-002	TGF-beta 1	R&D
302-B2-002	TGF-beta 2	R&D
243-B3-002	TGF-beta 3	R&D
100-21	TGF-beta1 (HEK293 cell derived)	Peprotech
100-35B	TGF-beta2 (HEK293 cell derived)	Peprotech
100-36E	TGF-beta3	Peprotech
288-TP-005	Thrombopoietin/Tpo	R&D
310-23	TL-1A	Peprotech
1319-TL-010	TL1A/TNFSF15	R&D
160-01	TLR-3 (HEK293 cell derived)	Peprotech
300-01A	TNF-alpha	Peprotech
300-01B	TNF-beta	Peprotech

300-18	TPO	Peprotech
375-TL-010	TRAIL/TNFSF10	R&D
390-TN-010	TRANCE/TNFSF11/RANK L	R&D
1548-TR-100	TROY/TNFRSF19	R&D
120-09	TSG	Peprotech
300-62	TSLP	Peprotech
310-06	TWEAK	Peprotech
310-21	TWEAK Receptor	Peprotech
150-16	VAP-1 (CHO cell derived)	Peprotech
130-11	Vaspin	Peprotech
150-04	VCAM-1 (HEK293 cell derived)	Peprotech
2347-VE-025	VEGF	R&D
100-20A	VEGF-A (121 a.a.)	Peprotech
100-20	VEGF-A (165 a.a.)	Peprotech
100-20B	VEGF-B	Peprotech
100-20C	VEGF-C (HEK293 cell derived)	Peprotech
100-20D	VEGF-D (HEK293 cell derived)	Peprotech
130-09	Visfatin	Peprotech
1341-WF-050	WIF-1	R&D
120-18	WISP-1	Peprotech
120-20	WISP-3	Peprotech
120-17	WNT-1	Peprotech
6179-WN-010	Wnt-11	R&D
5036-WN-010	Wnt-3a	R&D
6076-WN-005	Wnt-4	R&D
645-WN-010	Wnt-5a	R&D
120-31	WNT-7A (HEK 293 cell derived)	Peprotech
695-LT-025	XCL1/Lymphotactin	R&D

Supplemental Table 2: Table EC50 values from 12 colorectal cancer cell lines treated with a dose titration of GDC-0941 with and without 50 ng/ml of EGF and assayed for viability using CellTiter-Glo 96 hrs after dosing.

Cell line	GDC-0941 EC50(uM)	GDC-0941 +EGF (50ng/ml), EC50 (uM)	Fold increase
DLD1	0.455	0.795	1.75
HCT116	0.49	0.475	0.97
LS 174	0.767	1.73	2.26
LS 180	0.532	0.943	1.77
SW48	0.0217	0.0911	4.20
SW620	0.167	0.215	1.29
SW837	0.073	0.441	6.04

Supplemental Table 3: Table EC50 values from SW48 and SW48 H1047R parental and GDC-0941 resistant clones treated with a dose escalation of the PI3K inhibitor GDC-0980 and assayed for viability using CellTiter-Glo 96 hrs after dosing.

Cell line	GDC-0980 EC50(uM)
SW48	0.1684
SW48-R (2F)	1.84
SW48-R (2G)	1.18
SW48 H1047R	0.136
SW48 H1047R-R (10A)	1.658
SW48 H1047R-R (10B)	1.283
